# “We did not know what was wrong”—Barriers along the care cascade among hospitalized adolescents with HIV in Gaborone, Botswana

**DOI:** 10.1371/journal.pone.0195372

**Published:** 2018-04-09

**Authors:** Leslie A. Enane, Keboletse Mokete, Dipesalema Joel, Rahul Daimari, Ontibile Tshume, Gabriel Anabwani, Loeto Mazhani, Andrew P. Steenhoff, Elizabeth D. Lowenthal

**Affiliations:** 1 Division of Infectious Diseases, The Children’s Hospital of Philadelphia, Philadelphia, Pennsylvania, United States of America; 2 Department of Pediatrics, The Children’s Hospital of Philadelphia, Philadelphia, Pennsylvania, United States of America; 3 Ryan White Center for Pediatric Infectious Disease and Global Health, Indiana University School of Medicine, Indianapolis, Indiana, United States of America; 4 Botswana-UPenn Partnership, Gaborone, Botswana; 5 Botswana-Baylor Children’s Clinical Centre of Excellence, Gaborone, Botswana; 6 Faculty of Medicine, University of Botswana, Gaborone, Botswana; 7 Department of Pediatrics, Baylor College of Medicine, Houston, Texas, United States of America; 8 Center for Global Health, The Children’s Hospital of Philadelphia, Philadelphia, Pennsylvania, United States of America; 9 Perelman School of Medicine, University of Pennsylvania, Philadelphia, Pennsylvania, United States of America; 10 Center for Clinical Epidemiology and Biostatistics, Perelman School of Medicine, University of Pennsylvania, Philadelphia, Pennsylvania, United States of America; The Ohio State University, UNITED STATES

## Abstract

High mortality among adolescents with HIV reflects delays and failures in the care cascade. We sought to elucidate critical missed opportunities and barriers to care among adolescents hospitalized with HIV at Botswana’s tertiary referral hospital. We enrolled all HIV-infected adolescents (aged 10–19 years) hospitalized with any diagnosis other than pregnancy from July 2015 to January 2016. Medical records were reviewed for clinical variables and past engagement in care. Semi-structured interviews of the adolescents (when feasible) and their caregivers explored delays and barriers to care. Twenty-one eligible adolescents were identified and 15 were enrolled. All but one were WHO Clinical Stage 3 or 4. Barriers to diagnosis included lack of awareness about perinatal HIV infection, illness or death of the mother, and fear of discrimination. Barriers to adherence to antiretroviral therapy included nondisclosure, isolation, and mental health concerns. The number of hospitalized HIV-infected adolescents was lower than expected. However, among those hospitalized, the lack of timely diagnosis and subsequent gaps in the care cascade elucidated opportunities to improve outcomes and quality of life for this vulnerable group.

## Introduction

Worldwide, adolescent AIDS-related deaths are increasing, despite improvements in access to antiretroviral therapy (ART) [1]. From 2005–2012, while AIDS-related deaths declined by 30% in all other age groups, AIDS-related deaths in adolescents increased by 50% [2]. HIV/AIDS is a leading cause of death among adolescents globally, particularly in sub-Saharan Africa [3].

Mortality increases are particularly striking among 10–14 year-old adolescents with HIV, reflecting failures to diagnose perinatally infected children and to successfully engage them in care early in the course of their illnesses. Gaps have been described throughout the adolescent HIV care cascade—at HIV diagnosis, linkage to and retention in care, and ART adherence and suppression of viral load [4]. There is an urgent need to identify strategies to help adolescents make the same morbidity and mortality gains seen in other age groups.

Botswana is among the countries with the highest HIV prevalence in the world, with 21.9% of the adult population living with HIV [5]. Botswana has been a pioneer in responding to the HIV/AIDS epidemic; Botswana introduced PMTCT services in 1999, and in 2002 was the first sub-Saharan African country to provide universal free ART to citizens [6]. Botswana is now approaching 90-90-90 targets; however, successes are not shared equally across age groups. While it is estimated that 84% of Botswana’s adults living with HIV are receiving ART, only 60% of Botswana’s children (under 15 years) living with HIV are on ART [5]. Age-disaggregated outcome data are not available for the approximately 17,000 adolescents living with HIV in Botswana [5].

By investigating delays in the HIV care cascade among hospitalized adolescents with HIV in this high-prevalence setting, we aimed to examine the full spectrum of missed opportunities for HIV care in those most vulnerable to poor outcomes.

## Methods

### Setting

This study was conducted between July 2015 and January 2016 at Princess Marina Hospital (PMH) in Gaborone, Botswana, the country’s main government referral hospital. PMH is located adjacent to the Botswana-Baylor Children’s Clinical Centre of Excellence, which has provided comprehensive HIV care to thousands of children and adolescents since 2003, and provides adolescent-centered services, such as a Teen Club support group. HIV testing is indicated for all hospitalized children and adolescents with unknown HIV status, per Botswana National HIV & AIDS Guidelines [7].

### Data collection

The study nurse and other research team members consulted with ward medical teams to identify all adolescents (ages 10 to 19) with HIV hospitalized at PMH for any admission diagnosis other than pregnancy over a 6-month period. Adolescents admitted for any admission diagnosis to the medical, pediatric, surgical, oncology, or intensive care wards were included. Pregnant patients were excluded because the intent was to understand reasons for delayed access to care, and pregnant patients with HIV represent mainly those with more recent behaviorally-acquired infections in this context [5,8]. Prenatal care (PNC) uptake is high and 92.8% of mothers in Botswana’s PNC program were tested for HIV in their last pregnancy [8]. Furthermore, hospitalizations of pregnant adolescents in this setting are primarily for labor and delivery, or obstetric complications, rather than complications of HIV, which were our primary interest. Adolescent HIV deaths are roughly equal among males and females, and highest among 10–14 year-olds, arguing against pregnancy being a major driver of adolescent HIV deaths [2,9].

We developed semi-structured interview guides which begin with open-ended questions querying the adolescents’ prior engagement in care (S1 File). Sequential questions determined if critical failures or delays occurred in the diagnosis and treatment cascade. Probes investigated barriers to care at each stage of the cascade where critical challenges or failures occurred. The acceptability of specific proposed interventions to improve care at each stage was explored. Separate interview guides were used for disclosed and non-disclosed adolescents, and for caregivers. Guides for non-disclosed adolescents did not mention HIV, but asked generally about barriers to medical care for adolescents, as well as any challenges with adherence to medicines and clinic visits.

Eligible adolescents and their caregivers were approached with information about the study and to request informed consent/assent. Strict precautions were taken to prevent inadvertent disclosure of HIV status throughout the informed consent process and study procedures. This included avoidance of inadvertent disclosure to young adolescents who were not aware of their own status by avoiding use of the term HIV in any assent documents or in the interview guide for non-disclosed adolescents. Adolescents were presumed to be non-disclosed unless their primary caregiver confirmed that the adolescent was aware that he or she had HIV infection. Caregivers and adolescents (when medically stable) were interviewed separately in a confidential setting near the hospital ward. Interviews were conducted in Setswana by a bilingual research nurse with extensive experience caring for children and adolescents with HIV and training in interview techniques. Clinical and demographic variables were abstracted from medical records onto paper forms, and uploaded into a secure REDCap database [10]. A master list of study numbers and identifying information was kept separate from the de-identified study data used for analysis. Study materials were kept in a locked cabinet in a secured office where only the study team had access.

### Data analysis

Clinical and demographic data were analyzed using standard descriptive statistics, using Stata/SE 14.1 (StataCorp, College Station, Texas, USA). Interviews were audio-recorded, transcribed, and translated into English. The first and senior authors reviewed all transcripts and relayed queries to the study nurse, who contacted participants with follow-up questions when clarification was needed. Transcripts were coded and analyzed through thematic analysis. Initial codes were organized according to stage of the care cascade addressed by the participants, as well as hypothesized factors impacting the care cascade. Through reading and re-reading of the data, the coding structure was augmented with themes that emerged from the data [11,12]. NVivo 11 analysis software (QSR International Pty Ltd, Melbourne, Australia) was used to organize and analyze the data.

### Ethics statement

All procedures performed were in accordance with the principles expressed in the 1964 Helsinki Declaration. Written informed consent was obtained from all adolescents over age 18, and from parents/guardians of adolescent minors. Written assent was obtained from adolescent minor participants. Adolescents who were too ill to participate in assent procedures were included if their primary caregiver consented to review of the adolescent’s chart, and to be interviewed about the adolescent’s past engagement in care. The study protocol was approved by the institutional review boards of the University of Botswana, Princess Marina Hospital, the University of Pennsylvania, Children’s Hospital of Philadelphia, and the Health Research Development Committee at the Botswana Ministry of Health.

## Results

Over the 6-month study period, 21 adolescents meeting inclusion/exclusion criteria were hospitalized at PMH. Fifteen were enrolled in the study. One eligible adolescent died before enrollment and caregiver consent was withdrawn after another died. Two minors did not have a caregiver present who could consent to their participation and two declined consent/assent. Of all eligible adolescents, 4/21 (19.0%) died; among the adolescents enrolled in our study, 2/15 (13.3%) died during the hospitalization. Interviews were conducted with 13 adolescents and 15 adult caregivers, of whom 9 were mothers, 4 were sisters, one was an aunt, and one was a grandmother to the hospitalized adolescent.

The median age of the adolescents was 14.1 years (interquartile range [IQR 12.7–17.6]). Nine (60%) adolescents were 12–14 years old (Table 1). Median age at HIV diagnosis was 7.5 years (IQR 1.5–12.1 years). All were thought to be perinatally infected, given the age at diagnosis, clinical course, and family history. Two-thirds of patients were diagnosed with HIV after the age of 5 years (10/15, 66.7%), and a third after the age of 10 years (5/15, 33.3%). Of 10 patients with available labs from time of diagnosis, 3 (30.0%) had advanced immunodeficiency, and 7 (70.0%) had severe immunodeficiency [13]. 14/15 (93.3%) of patients were WHO Clinical Stage 3 or 4 [13].

**Table 1:** Characteristics of enrolled adolescents.

Characteristic	N = 15
Age at admission, years	Median 14.1, IQR (12.7–17.6)
12–14	9 (60.0)
15–17	4 (26.7)
18–20	2 (13.3)
Female sex	5 (33.3)
Orphan status	
Both parents deceased	2 (13.3)
Mother deceased	3 (20.0)
Father deceased or unknown vital status	7 (46.7)
Both parents living	7 (46.7)
Left education or repeated school year due to illness	10 (66.7)
-Prior to HIV diagnosis	3 (20.0)
Age at diagnosis, years	Median 7.5, IQR (1.5–12.1)
<1	2 (13.3)
1-<2	2 (13.3)
2–5	1 (6.7)
6–10	5 (33.3)
> 10	5 (33.3)
WHO Immune Stage at diagnosis (n = 10)	
HIV-associated immunodeficiency:	
None or not significant	0
Mild	0
Advanced	3 (30.0)
Severe	7 (70.0)
WHO Clinical Stage	
Primary HIV or Stage 1	0
Stage 2	1 (6.7)
Stage 3	7 (46.7)
Stage 4	7 (46.7)
Recent CD4 (n = 10)^a^	Median 367, IQR (65–973)
Recent VL available^b^	10 (66.7)
Recent VL undetectable	9 (90.0)
HIV status disclosed to adolescent^c^	9 (60.0)

^a^CD4 tested within the past 6 months prior to admission.

^b^Viral load tested within the past 6 months prior to admission.

^c^Median age at disclosure 11.8 years.

Diagnoses made on admission or during hospitalization were categorized by systems (Table 2). The majority of adolescents (9/15, 60.0%) were hospitalized for AIDS-related diagnoses. Pulmonary tuberculosis (TB) (5/15, 33.3%), anemia (5/15, 33.3%), severe malnutrition (4/15, 26.7%), and oncologic diagnoses (3/15, 20.0%) were noted in multiple patients. Two patients were admitted for elective surgical procedures, one of which was probably HIV-related (tenotomy needed due to early life encephalopathy).

**Table 2:** Diagnoses given on admission or during hospitalization per medical team^a^.

Admission / Hospital Diagnoses^b^	Patients (%)
AIDS-related diagnoses^c^	9 (60.0)
Pulmonary tuberculosis	5 (33.3)
Severe malnutrition or wasting	4 (26.7)
Oncologic	3 (20.0)
Squamous Cell Carcinoma	1 (6.7)
Metastatic pharyngeal carcinoma	1 (6.7)
Kaposi sarcoma	1 (6.7)
Cardiac	2 (13.3)
Congestive heart failure	1 (6.7)
Hypertrophic cardiomyopathy	1 (6.7)
Hematologic	6 (40.0)
Deep vein thrombosis	1 (6.7)
Anemia	5 (33.3)
Acute on chronic renal failure	1 (6.7)
Central nervous system	3 (20.0)
Cryptococcal Meningitis	1 (6.7)
Seizures	1 (6.7)
Cerebral Palsy	1 (6.7)
Dermatologic	2 (13.3)
Stevens-Johnson Syndrome	1 (6.7)
Herpes simplex virus infection	1 (6.7)
Drug reaction^d^	2 (13.3)
Elective surgical procedures	2 (13.3)

^a^Abbreviations: AIDS, acquired immune deficiency syndrome.

^b^Note that some patients carried multiple diagnoses.

^c^Including TB, severe malnutrition, cryptococcal meningitis, HSV infection, and AIDS-related malignancies.

^d^AZT-induced anemia (1) and ART-related Stevens-Johnson Syndrome (1).

Only the two patients admitted for elective surgical procedures were diagnosed in infancy. All other adolescents had experienced delays in HIV diagnosis (Table 3). While delays were common, only one patient was diagnosed during the current hospitalization, at the age of 12. Significant challenges to retention in care were identified, with 3/14 previously diagnosed adolescents having been out of care as much as several years. Of 13 patients on ART, 6 reported significant adherence difficulties.

**Table 3:** Missed opportunities in the HIV care cascade^a^.

Challenges in HIV care over disease course	Patients (%)
**Diagnosis**	
Delayed or failed^b^	13/15 (86.7)
Diagnosed prior to hospitalization	14/15 (93.3)
**Linkage**	
Delayed after diagnosis	0/14 (0)
Linked prior to hospitalization	14/15 (93.3)
**Retention**	
Not retained in course of care	3/14 (21.4)
Retained in care prior to hospitalization	13/15 (86.7)
**ART**	
Delayed initiation^c^	0/13
Adherence difficulties^d^	6/13 (46.2)
On ART prior to hospitalization	13/15 (86.7)
Recent VL done and undetectable^e^	9/15 (60.0)

^a^Abbreviations: HIV, human immunodeficiency virus; ART, antiretroviral therapy; VL, viral load testing.

^b^Diagnosis outside of infancy considered delayed.

^c^Among those in care.

^d^Based on interview responses (caregiver and/or adolescent).

^e^Viral load tested within the past 6 months prior to admission.

Specific barriers and missed opportunities for care were explored for each stage of the cascade where adolescents experienced significant delays or challenges (Table 4). Many barriers presented significant challenges at multiple stages of the cascade. Barriers related to diagnosis and adherence were most common and are presented in-depth.

**Table 4:** Key themes relating to missed opportunities, barriers to care, and areas for intervention along the HIV care cascade^a^.

Key Themes	Relevant Stage(s) of the Cascade	Illustrative quotes
**Missed opportunities for diagnosis**		
Multiple illnesses or hospitalizations prior to diagnosis	Diagnosis	“He started [becoming sick] when he was seven years old. He had chronic cough and he was losing weight slowly and slowly. He was admitted twice at [primary hospital] due to chronic cough which was blood-stained, and they were unable to diagnose TB.”–*Mother of a 15 year-old male*
		“He had diarrhea and it was before we knew his status, he used to go [to the clinic] for rehydration. [After three months] they saw that it was not becoming better, every two weeks he was having diarrhea, then they suggested that he be tested for HIV.”–*Sister of a 17 year-old male*
Withdrawn from school due to illness, prior to diagnosis	Diagnosis	“She stopped [school] due to ill health. [She was suffering from] dizziness, inability to walk, vomiting, and diarrhea.”–*Mother of a 14 year-old female*
		“He repeated [the school year] due to ill health. We were going to the local clinic time and again, not knowing what was wrong with him.”–*Grandmother of a 12 year-old male*
Delayed or missed HIV diagnosis of the mother	Diagnosis	“Healthcare workers were advising me about testing [the child]. But because I had not tested myself I did not believe that my child could have contracted the disease.”–*Mother of a 12 year-old female*
		“When I was pregnant with him I had tested negative. I didn’t have the virus, or maybe it was hidden somewhere in my blood, I don’t know. So I breastfed him. … The child was tested [at age 5] and was found to have HIV. So I also tested.”–*Mother of a 15 year-old male*
**Barriers to care**		
Lack of awareness of possibility of late presentation of perinatal HIV	Diagnosis	“My mother did not know that she was supposed to test the child because she thought she was the only one infected. And also the child grew up well-nourished and breastfed with no problems. I think that’s what delayed her thinking to test the child.”*–Sister of a 19 year-old female*
Illness or death of the mother	Diagnosis, Linkage, Retention, Adherence	“I have no idea [why mother didn’t bring me for testing earlier], I just know that she was very sick and unable to do anything for herself. And when she was still alive I didn’t have problems with recurrent sores. After she passed away, they troubled me a lot.”– *19 year-old female*
		“For this child to be tested it was tough. The child was very sick… as a result of late diagnosis… The elders in the family were encouraging my sister to test the child. I think she was in denial… but after some time when the child was not improving, she took the decision to test the child.”–*Sister of a 17 year-old male*
		“My mother became very sick. So, there was no one to bring me for hospital checkups.”– *13 year-old male*
Fear of knowing the result of HIV testing	Diagnosis	“Problem with young people—it’s like they are scared of testing and I don’t know why because there are some ARVs.”–*Grandmother of a 12 year-old male*
		“I was scared [to test my child]. Those were the years when it was still scary. The fact that it was said, ‘HIV is dangerous and it kills,’ was scary.”–*Mother of a 12 year-old female*
Consent issues	Diagnosis	“Last year at [local clinic] the doctor had referred me to [a different clinic] to have them test the adolescent. So, I asked his uncle to accompany him as I [the grandmother] had signed the consent for HIV testing. When he got there, they told him they want the biological mother. I was shocked when I heard that as his mother was nowhere to be found. They had collected other blood tests but not the one for HIV. I was not happy because I had wanted this child to be tested for HIV. … The boy was sick.”–*Grandmother of a 12 year-old male*
Lack of disclosure to family members	Diagnosis, Linkage, Retention, Adherence	“She was very sick … On discharge she was still not well and I brought her here [to the hospital] and we were going to [the local clinic] where she was also presenting with diarrhea and they asked me if the child has been tested. Because I didn’t know, I said ‘no’ and they told me to take her for HIV testing and bring back the results. But before I left they picked it up from the medical cards that the child had been tested long ago and was started on treatment which was stopped at some point, and they referred me to [HIV treatment clinic].”–*Aunt of a 14 year-old female*
		“His mother is the one who was living with him then, and I didn’t ask if the child was tested for HIV; she also didn’t mention anything. When she told us that he was diagnosed with TB we never thought of anything else. We were happy that at least there was a diagnosis.”–*Grandmother of a 12 year-old male*
Need for family support		“[Family support] is important because I have my siblings and they have to know about this child’s life, what is going on so that if maybe I am not feeling well there will be someone to continue my child’s care, how to handle her, how to give medications. All my siblings they don’t even know where my child goes for checkups. Even when I say, ‘my child is sick,’ they don’t get it, and it’s not good as I also need support and love from them. … It’s like it’s my problem alone. So I ask myself, is it because she has HIV now others do not want to get involved? So if they have been explained, taught, they will feel they have to participate in the child’s care.”–*Mother of a 12 year-old female*
Stigma and fear of discrimination	Diagnosis, Linkage, Retention, Adherence	“His schoolmates saw him in a queue at [the clinic] for his refills and at school they asked him what he was doing there, because they know that that side is for people living with HIV. …So he started to say he does not want to take his ARVs.”–*Mother of a 15 year-old male*
		“I think her reason for not taking medications could be [because] she likes to go around a lot visiting friends. Maybe when she is with friends she is not free to take medications because she does not forget them. She carries the medications with her.”–*Sister of a 19 year-old female*
Mobility, living with other family or peers	Diagnosis, Linkage, Retention, Adherence	“She once disappeared from home and we didn’t know where she was and we were not sure if she was going for checkups. Her cards were here with us.”–*Sister of a 19 year-old female*
		“I cannot live with my children alone. At some point they should be free to visit their grandparents. So, they should know what kind of children they are, what time they take their medications, and how do they take them.”–*Mother of a 12 year-old male*
Non-disclosure to adolescents	Adherence	“I sometimes skip doses … because I want to know why I am taking these medications.”– *12 year-old male*
Need for increased adherence support	Adherence	“His main problem is he is in serious denial. He does not want to accept his status. He says he is not sick; he is fit. We talk to him all the time about the importance of taking medications, and his response will be, ‘I am fine, I don’t need pills.’… They used to have counseling sessions with him, and he will agree as if he does understand in front of them, but when we get home he changes. … He keeps asking will he ever stop these medications.”–*Mother of an 18 year-old male*
		“She sometimes stops taking [her medications]. Even if sometimes when I ask her if she has taken them, she will say ‘yes,’ but I will discover that she has spit them on her T-shirt.”–*Sister of a 19 year-old female*
Family poverty	Retention, Adherence	“To tell the truth, if there is not food at home I won’t manage to take my medications. … Even transport money it’s a challenge … sometimes we borrow from neighbors and pay them back.”– *19 year-old female*
Isolation and mental health concerns	Retention, Adherence	“What I need for her is counseling … because sometimes she says she is asking herself why she is always the one very sick at home, what has she done wrong. It shows something is bothering her. It’s on and off. She speaks like that especially when she is very sick, sometimes she will even be refusing to go to the clinic, saying she wants to be left to die.”–*Sister of a 19 year-old female*
		“I think [a peer support group] is good for my adolescent as it can teach him something good, so that if he is with other people he should not give up and think he is the only one with HIV. I have realized that when he is with his age mates he looks like someone without some confidence, thinking, ‘I have the virus,’ and I think that contributes to his poor adherence.”–*Sister of a 17 year-old male*
Lack of adolescent-centered services	Retention, Adherence	“He needs to socialize, he asks a lot of questions like, ‘are there people of my age with the same status?’ Like when he came for admission he asked me, ‘are there a lot of people where I am going to be admitted?’ I said ‘ yes’ and he said, ‘are there my age mates there?’ So when he found out that in the ward there were a lot of adults and no age mates he did not like it at all.”–*Sister of a 17 year-old male*
		“Where we go for HIV care there is nothing like the teen support groups for the chronically sick ones. We just go for the doctor’s consultation and do refills then go home. Even if we speak with him at home now I feel he needs such peer support.”–*Mother of an 18 year-old male*
**Facilitators to care**		
Adolescent disclosure		“We have talked and he has really accepted his status since the time he saw the test results. It’s us who were worried, but him he was just fine.”–*Sister of a 17 year-old male*
		“She has accepted it, though it’s not something that she is happy about. She has sort of accepted and is a forgiving type, she understands. When you explain something to her when she asks, she understands.” [Mother tearful.]–*Mother of a 14 year-old female*
Family disclosure and support	Diagnosis, Linkage, Retention, Adherence	“I had not told anyone about my child’s status at home for fear of being discriminated against, even though they knew my status. … The family at home they all know now. I told them and they don’t discriminate against him. Because I may get held up and I am able to send him with his elder sister for checkups or any other family members. We discussed the child’s illness at home and they accepted him and support him well.”–*Mother of a 13 year-old male*
Family supervision	Retention, Adherence	“Before we used to just tell him to go and drink medications because it’s time and we realized he was not taking them sometimes. Now we monitor him even if he comes home late we make sure he takes his medications.”–*Sister of a 17 year-old male*
Mental health support, counseling	Diagnosis, Linkage, Retention, Adherence	“The only [intervention] that I am thinking about is continuous counselling and support. As parents we are offering it but a different person will help as well.”–*Mother of a 14 year-old female*
**Proposed interventions**		
Enhanced personal contacts	Retention, Adherence	“It’s a good program, because if you are left with seven days and you are reminded to come to the hospital it’s a very good thing, it gives the caregiver some wisdom.”–*Sister of a 17 year-old male*
		“It can be used for others, but as for me and my family, we don’t need it because I never forget the checkup dates for both of us.”–*Mother of a 15 year-old male*
Clinical decision support	Retention, Adherence	“It’s important to call as it shows we are working together in the child’s care, as nurses and caregivers. It also shows health workers do care about our children’s health.”–*Mother of a 12 year-old female*
		“[It would be] good for me and my family, because I lost an older sibling who was not adhering to treatment. He had moved away from home and after he passed away we found out that he was on HIV treatment but had long ago defaulted. So it will help to avoid losing lives.”–*Sister of a 17 year-old male*
Peer support	Retention, Adherence	“If we suffer from the same illness we become free with each other. For example, one will maybe say, ‘I don’t like Combivir it does not make me feel good,’ and the peer will respond, ‘but Combivir it’s good because it suppresses the virus.’ So they are able to encourage each other as peers.”–*Sister of a 19 year-old female*
		“[Joining a peer support group] will help me to understand that whether you are on medications or not you are still the same.”– *12 year-old male*
		“We will be in the same air together, and no one will be like saying, ‘this one has HIV and this one does not have,’ it will be just fun.”– *14 year-old female*
		“It will help to be free when I am with others.”– *13 year-old male*
Family therapy	Retention, Adherence	“I have an aunt who is also on ARVs. I once thought maybe my aunt could join us when we come for clinic checkups so that she discloses to the boy as part of encouragement; maybe he will see that he is not alone.”–*Sister of a 17 year-old male*
		“I am not sure because it’s still between me and her father, my mother and uncles from my side. Because I am not yet married to her father we have not disclosed to her father’s relatives. There is no way they can [participate in] this kind of support.”–*Mother of a 12 year-old female*
Cell phone reminders	Retention, Adherence	“Yes it will help me to get reminders because sometimes I am away from home playing. So when it rings I will know that I have to come home.”– *17 year-old male*
		“I think it can benefit both even those who do well in treatment and checkups as people can forget but not intentionally.”–*Sister of a 17 year-old male*
		“Myself I use the cellphone reminder for medication time. She also knows if its time and I am outside and the alarm rings she tells me. The reminder is good for both the parent and the adolescent to work together.”–*Mother of a 12 year-old female*
		“No, I think it’s all about commitment as a parent. To always be reminded will be like as the caregiver you don’t care, I don’t support the idea.”–*Mother of a 13 year-old male*

^a^Abbreviations: HIV, human immunodeficiency virus; TB, tuberculosis; ARVs, antiretroviral medications.

### Missed opportunities for diagnosis

Presenting frequently to clinics or hospitals without receiving an HIV test was reported by many. Chronic illnesses included cough, pneumonia, TB, diarrhea, ear infections, rashes, oral ulcers, and wasting. In a few cases, the child had to withdraw from school due to severe illness, but had not yet been tested for HIV. In some, failure to diagnose the mother may have contributed to delayed diagnosis of the child.

### Barriers to HIV diagnosis, linkage, and retention in care

#### Lack of awareness

Caregivers frequently discussed their past lack of knowledge of the possibility of undiagnosed perinatal HIV infection in their children, or remarked that they “did not believe” that the child could be infected. Some noted that the child “grew up well-nourished” or “was doing fine” prior to becoming ill. When the child did become ill, multiple caregivers remarked that “it never crossed [their] mind” or that they “never thought [that the child could have HIV].” In some cases, the mother herself had not yet been diagnosed—one mother reported, “even myself I had not tested.”

#### Illness or death of the mother

Severe illness or death of the child’s mother was a common contributor to delayed diagnosis or failure to retain a child in care when “there was no one to bring [the child] to clinic”. Challenges in adherence and retention for mothers–“her mother stopped treatment and is now very sick”–contributed to delays in children’s care. In these situations, other family members needed to take on the medical care of the child, including the decision to test for HIV.

#### Lack of disclosure to other family members

Lack of disclosure of the child’s status to other family members was an important barrier to diagnosis and retention in care, particularly if the mother was ill, or if the child was living with family members who were not aware of the child’s status. Two adolescents had several-year gaps in care after diagnosis, due to severe illness of the mother and lack of disclosure of the child’s status to other family members who potentially could have brought the child for care.

I was very sick and bedridden. I had once come to clinic after he was diagnosed, for initiation [of ART], but I stopped as there was no one to bring him. I had not told anyone about my child’s status at home, for fear of being discriminated against, even though they knew my status.

—*Mother of a 13 year-old male*

#### Stigma and fear of discrimination

Stigma and fear of discrimination were barriers to diagnosis and care at all stages of the cascade. Caregivers expressed fear of discrimination, even from close family members, as in the preceding example. They reported that stigma was also acutely experienced by adolescents, particularly in their interactions with peers.

It hurts him; he does not like it. Especially when you speak with a raised voice, telling him he has the sickness, his perception is maybe other people are listening.

—*Sister of a 17 year-old male*

We need to meet with health professionals to continue talking about this illness, so that the child understands that this is an illness like any other; she does not differ from others.

—*Sister of a 19 year-old female*

### Adherence challenges

Adherence challenges were commonly described among adolescents on ART. Key barriers to adherence included: lack of disclosure of HIV status to the adolescent or family members, stigma, isolation, and mental health concerns including para-suicidal behavior. (A sister of a 19 year-old female reported, “sometimes she will even be refusing to go to the clinic, saying she wants to be left to die.”) For those adolescents who had been told their HIV status, adolescents and caregivers spoke of both the challenges and breakthroughs for these youth in “accepting themselves,” or coming to terms with the diagnosis.

### Potential interventions

Participants were asked about specific proposed interventions at each stage along the cascade where challenges were faced (Table 4). Peer support was currently available to some, but not all, of the disclosed adolescents, and was seen as a particularly vital intervention to support adolescent mental health, confidence, education about HIV, and adherence. Adolescents described the peer group setting as a means to escape isolation and stigma—to “be in the same air together”–to “be free with each other.” As one 12 year-old male stated, “[the groups help us] to understand that whether you are on medications or not you are still the same.” Unfortunately, many adolescents were not able to benefit from a peer group due to being unaware of their HIV status or receiving care at sites lacking peer groups.

Regular reminders from personal contacts were seen by some as helpful, though others felt that visit reminders were unnecessary for themselves. Similarly, cell phone reminders (of medication schedule or of clinic visits) were generally not perceived as being as helpful as phone calls from clinic staff in the case of missed appointments or concerning lab results. Such outreach was seen as evidence of a strong relationship of the clinic staff to the adolescent. Multiple caregivers noted, “it shows that they care.”

Family therapy interventions were generally viewed favorably. Several caregivers noted that the involvement of other family members could provide additional encouragement and support to the adolescent. A few caregivers and adolescents expressed concerns relating to disclosure of the adolescent’s status to close family members–“I just don’t want them to know.” Family interventions may be challenged by limited disclosure within the family.

Caregivers and adolescents offered additional unprompted insights into key facilitators to adolescent care, including adolescent disclosure support, family disclosure and involvement, and adolescent mental health services and counseling (Table 4). The need for additional adolescent counseling was noted by multiple caregivers, as they described significant isolation and mental health concerns among their adolescents. The importance of disclosure to the adolescent as well as to trusted family members were recognized as being critical to the discussed interventions.

## Discussion

Significant delay in HIV diagnosis emerged as the most consistent challenge in the care cascade in this study of hospitalized adolescents with HIV infection. The median age at HIV diagnosis (7.5 years) is older than it was once thought that children could survive with HIV untreated [14], and a third were diagnosed in adolescence. While long-term survival of perinatally infected adolescents is increasingly recognized among clinicians and researchers, most laypeople may not know that these children could potentially survive into adolescence untreated [15]. Few of these adolescents were born in the pre-ART era, when more than half died in the first 2 years of life [16]. Many improvements in pediatric HIV diagnosis have occurred since the time these adolescents were infants, with widespread availability of early infant diagnostic services and improved implementation of “opt out” testing. However, there has been insufficient emphasis on HIV diagnosis of young children in healthcare settings outside of the PMTCT context [17]. Most adolescents in this study presented to clinics or were hospitalized on several occasions, before being tested for HIV. For some, HIV testing was reportedly not offered despite their manifestations of HIV-related illnesses. For others, there was hesitancy on the part of the caregivers to test. Clinical and public health leaders in high HIV-burden settings should eliminate barriers to universal pediatric testing. This should include efforts to increase awareness among health care workers and lay people about the possibility of survival into older childhood and adolescence with perinatally acquired HIV. Other efforts to increase uptake of pediatric HIV testing in clinical settings, and to support caregivers through counselling and stigma-reduction interventions, should be pursued.

Late presentation of perinatal HIV infection is associated with profound immunosuppression and advanced disease [14]. Perinatally infected adolescents who do not initiate treatment until after long-standing, advanced immunosuppression are medically vulnerable [18]. Prolonged untreated HIV infection contributes to chronic inflammation, impaired immune recovery, neurologic and developmental pathology, and the development of opportunistic infections and malignancies. The fact that HIV is the leading cause of death among 10–14 year-olds worldwide suggests that we have failed to find and adequately treat far too many children before they develop profound illness [19]. The high mortality (19.0%) among adolescents with HIV in our cohort is consistent with that observed among children and adults hospitalized with HIV globally and in the African region [20]. There are very limited data around causes of hospitalization and mortality for adolescents with HIV infection; in our series, AIDS-related diagnoses were frequent, as has been observed among children and adults with HIV globally in the ART era [20].

Once diagnosed and linked to care, disengagement of children and adolescents is a complex challenge. A few adolescents in this series had become disengaged soon after diagnosis, and were out of care for years prior to hospitalization with advanced illness. The challenge of early disengagement from HIV care has been described in children in Botswana, and in studies of youth and adults elsewhere in sub-Saharan Africa [21–24]. Prior research has demonstrated the negative impacts of stigma, discrimination, and fear of disclosure on adult retention in HIV care [25]. In this series, maternal illness, stigma, and lack of disclosure to close family members influenced poor retention. Further research is needed to define barriers and facilitators to long-term retention for children and adolescents in high-prevalence settings. In addition, interventions to minimize disengagement of children and adolescents and to track and re-engage those who are lost to follow-up should be included in HIV care programs.

Stigma played a central role in breakdowns in the care cascade, and impacted adolescent care through pervasive effects on disclosure to the adolescent and to family. Family relationships, engagement with care, experience of isolation, and adherence to ART were also directly impacted by stigma. While stigma significantly complicates care for all persons living with HIV, perinatally infected adolescents have a particularly unique and challenging experience of stigma [26,27]. These adolescents transition from dependence on caregivers (who may themselves be facing severe illness, mental health issues, poverty, stigma, and isolation); to learning their status; coping with illness or death of family members; navigating peer relationships; and developing autonomy in care; all while undergoing the developmental transition of adolescence and into young adulthood. Interventions to reduce stigma and to mitigate the experience of stigma for adolescents—by strengthening family, peer, and community support—are urgently needed, and will be critical to improving adolescent HIV outcomes in the care cascade [26–29].

Problems adhering to ART were common in this study of hospitalized adolescents, and daunting adherence challenges in this age group are well documented [18,30,31]. It is important to recognize that in Gaborone, where pioneering adolescent-centered services are available to support adherence, adherence nevertheless remains a critical and complex challenge. Participants in this series were enthusiastic about participation in a peer group to reduce social isolation and improve HIV education and ART adherence. Many of the adolescents in this series were not receiving adolescent-centered services, such as engagement in a peer support group, either because their status was not yet disclosed to them, or because they were enrolled at clinics that did not provide these and other youth-friendly care services. Indeed adolescent-centered HIV care services remain an exception to the rule, and are not widely available in most settings [18]. For those engaged at clinics providing such services, some continued to have complex difficulties adhering to therapy, including mental health needs requiring ongoing counseling and support. The current evidence base for interventions to improve adolescent adherence is severely limited [32]. Studies of developmentally-appropriate approaches to improve adolescent adherence to ART are critically needed.

Despite the gaps in care and advanced illness experienced by the adolescents in this series, the number of adolescents identified at the tertiary referral hospital in Botswana over a 6-month period was unexpectedly small. This finding contrasts with alarming recent estimates that 100 adolescents are dying daily from HIV in sub-Saharan Africa [33]. While the HIV prevalence in Botswana is high, with its small population, the approximately 17,000 HIV-infected adolescents in Botswana make up less than 1% of the global population of adolescents with HIV, and an even smaller proportion of global adolescent HIV deaths, with fewer than 500 deaths annually [5]. PMH serves as both the major referral hospital for the country and the primary hospital for the capital city. However, our study may under-represent the adolescent epidemic in Botswana if adolescents with HIV were predominantly admitted to primary hospitals other than PMH or died in the communities. It’s worth noting that chronically ill patients in Botswana may be brought back to their ancestral villages for end-of-life care [34]. It is also possible that the low number of admissions reflects some relative successes of the pediatric HIV treatment program in Botswana, which may not be representative of the broader adolescent HIV epidemic in sub-Saharan Africa.

A further limitation of our study was our reliance on interviews for self-report of adherence challenges, and for recollection of delays and challenges earlier in the adolescent’s history. Recall bias may have minimized some barriers while over-emphasizing others. The breadth of our semi-structured interview was designed to ascertain the full scale of missed opportunities and challenges along the HIV care cascade for adolescents. However, this did not allow for exhaustive exploration of all specific barriers or potential interventions. For example, targeted interventions to support family disclosure and to mitigate mental health concerns were discussed with all participants, but not comprehensively evaluated by this study. Finally, by excluding pregnant adolescents whom we presumed would be admitted primarily for obstetric care and more likely to have behaviorally acquired HIV, we were not able to evaluate gaps in the care cascade prior to hospitalization with any HIV-related complications in this group. These are important areas for future investigations.

## Conclusion

These data highlight the failure of timely diagnosis of pediatric HIV infection among adolescents at the highest risk of poor HIV treatment outcomes. More work is still needed to address pervasive stigma, to study interventions for adherence and retention, to support disclosure to both adolescents and their close family members when needed, and to raise awareness of late presentation of perinatal HIV infection. Greater access to disclosure support, peer groups, adherence support, and mental health care is needed.

## Supporting information

S1 File Interview guides. Interview guides used for caregivers, disclosed adolescents, and non-disclosed adolescents.
(PDF)
