# Supplementary material for: “We did not know what was wrong”—Barriers along the care cascade among hospitalized adolescents with HIV in Gaborone, Botswana
Source: PLoS One. 2018 Apr 9;13(4):e0195372. doi: 10.1371/journal.pone.0195372 (PMC5890999; doi:10.1371/journal.pone.0195372)
Supplement: S1 File — Interview guides used for caregivers, disclosed adolescents, and non-disclosed adolescents. (PDF) [file pone.0195372.s001.pdf]

PARENT Interview Guide

Section 1. Demographics

1.1 Relationship to the adolescent:

1.2 Highest level of education completed by the caregiver:

1.3 Orphan status of the patient: (maternal, paternal, both, neither)

1.4 Parent's current occupation (month prior to hospitalization): (part-time work, full-time work, and/or going to school, informal labor only, too ill to go to school or work, other)

1.5 Patient's last completed school grade:

1.6 Did your child ever have to either repeat a year of school or to stop attending school because (he/she) was frequently ill?
(If yes, provide details.
When did these illnesses occur? What were your child's symptoms? Was the child hospitalized? What were the reasons the child needed to postpone or stop their education?)

1.7 HIV status of biological mother of the patient (positive, negative, unknown)

1.8 Do you live in the same home as the patient?

1.9 Does anyone in the patient's home have HIV? (If yes, ask relation to patient)

1.10 What roles do you play in your child's HIV care?
(Probes: How often are you the one who brings the child to clinic appointments? How often are you the one who picks up their medication refills? How often do you supervise them taking medications? What do you do to supervise? (remind only, remind and give pills, actually watch dose taken))

1.11 How have you talked about HIV with your child?
(*In what ways have you talked about it? What do you think your child thinks about it?*
If HIV status not disclosed*: What have you said to your child about their illness?*
When do you plan to disclose their HIV diagnosis to them? How do you plan to do that?)

Section 2. HPI/PMHx

2.1 Why is your child in the hospital?
(Probe for details of what the caregiver understands about the adolescent's current illness or hospitalization.
If parent's answer differs from registry and chart information, probe for clarification.)

2.2 How long has your child been sick, or unwell?

(Probe for specifics about disease course:
Ask about symptoms, progression of illness since the child first became sick.
Where did the child go for care? (clinic / hospital / traditional healer)
What was done at these medical visits?)

2.3 Has your child ever been admitted to the hospital in the past?

(If so, when did the admission(s) occur?
What was/were the reason(s) for the admission(s)?
How long was the child in the hospital?
Did the child fully recover, or was the child persistently ill?
Was the child admitted frequently to the hospital?)

2.4 In the last year, did your child ever go to clinic because (he/she) was sick?

(If so, please tell me about that.
When did the child go to clinic?
What symptoms was the child having?
Specifically ask if there was any presentation with: flu-like symptoms, fever, decreased appetite, sore throat, malaise or feeling "unwell", rash, night sweats, sores, swollen or tender lymph nodes, muscle aches, and/or headache.
What care did the child receive?
Did the child recover? Or was the child persistently ill?)

<u>Section 3. HIV testing and follow-up history – All patients.</u>

3.1 Was your child ever tested for HIV prior to this hospitalization?

3.1b **How many times did the child present to care (to a clinic or hospital) before the child was tested for HIV**?

What were the reasons for presenting to care? (What symptoms or illnesses did the child have?)
Where did the child go for care?
Did you think the child's illness might be related to HIV?
Was your child ever offered an HIV test?
Did you ever ask for your child to be tested for HIV?

Review the adolescent's health card and clarify details of previous clinic visits or hospitalizations and illnesses. *Note if each episode was a clinic visit or hospitalization.*

<u>Month/Year</u>	***<u>Hospital or Clinic</u>***	***<u>Reason for Visit or Admission</u>***

(If NOT diagnosed until this admission, SKIP to section 4)

3.2 When was your child diagnosed?
(What illnesses was your child suffering from? What led to your child being tested? Where was the child tested?
Why do you think the child wasn't tested earlier?)

3.3 After your child was diagnosed, did (he/she) ever go to a clinic for HIV care?
(*If NOT connected to a treatment center, SKIP to section 5*)
How soon after the child was diagnosed did the child go to clinic for HIV care?
If there was a significant gap between diagnosis and linkage to care, why did this occur?
Where does the child go for care?
Has the child ever gone anywhere else for their care?
If so, why was there a change in where the child went for care?

3.4 When was the last time your child went to a clinic for HIV care?
(If MORE than 6 months ago SKIP to section 6; if LESS than 6 months ago, SKIP to section 7)
In the past, has your child ever stopped going to clinic for a period of time?
When was that? Why was the child unable to go to clinic during that time?
What helped the child get back to clinic?

Section 4. Not diagnosed.
4.1 Did you ever think about getting your child tested for HIV before this hospitalization?
(*Probes: Why, or why not?*
If you or the child's doctors thought about it, but did not test:
Why wasn't the child tested?
Do you know where you can go to get your child tested?
Would you feel comfortable going there to get the test?
Cost a barrier?
Stigma/fear of knowing a barrier?
Any other reasons you did not have your child tested?)

4.2 Is there anything that would have made you get your child tested for HIV earlier?

4.3 Do you have suggestions for how healthcare workers can help young people get tested before they get sick?

4.4 Where do you think tests should be offered?
(Probes:

What about in schools?
What about in people's homes through door to door testing?
What are some reasons this could be helpful?
What do you think might be some problems with this?)

END INTERVIEW HERE

<u>Section 5. Diagnosed but not linked to care.</u>
5.1 When your child was diagnosed with HIV, what were you told about getting HIV care?
(Probes:
Were you aware of your child's diagnosis?
Was care offered at the same place where your child was diagnosed?
Why didn't you or your family do what you were told to do to get HIV care?
Probes: transportation, convenience, expense, confidentiality, stigma, trust in medical system)

In some places, different things have been tried to help people with HIV get the care they need. I would like to tell you about these things and hear whether or not you think they would be helpful to adolescents like yours in Botswana?

5.2 <u>Extended Counseling:</u> In some places, there are long counseling sessions for people who just learned that they have HIV. The counseling sessions happen in the homes of people with HIV and happen every month for about 2 hours. They talk about lots of things related to HIV, including why medicines for HIV are important.
Do you think this type of counseling program would have been good for you and your family?
Why or why not?

5.3 <u>Case Management</u>: In some places, people called "case managers" meet with HIV+ people in whatever place the HIV+ person and their family are most comfortable. This might be in their homes or the clinic or anywhere else. They help them to connect to helpful resources such as support groups and clinics. They also help them to think about other ways to reach goals that they have in life.
Do you think this type of program would have been good for you and your family?
Why or why not?

5.4 Is there anything else that you think would have helped your child to link to care?
END INTERVIEW HERE

<u>Section 6. Linked but not retained in care.</u>
6.1 Why did your child stop going back to the clinic for HIV care?
(Probes: cost, family beliefs, patient-provider relationship)

In some places, different things have been tried to help people with HIV stay in care. I would like to tell you about these things and hear whether or not you think they would be helpful to other adolescents like yours in Botswana?

6.2 Enhanced Personal Contacts: In some places, there are people at the clinics who call patients between clinic visits to remind them about appointments. The patients meet these people when they come to their appointments. When patients miss appointments, they call them to help them figure out how they can get back to the clinic.
Do you think this type of program would have been good for you and your family?
Why or why not?

6.3 Clinical Decision Support: In some places, clinic staff don't call patients with reminders, but do call if there is a problem such as a missed appointment.
Do you think this type of program would have been good for you and your family?
Why or why not?

6.4 Peer Support: In some places, young people with HIV are linked to peers with HIV through support groups or other activities.
Do you think this type of program would have been good for your child?
Why or why not?

6.5 Family Therapy: For other illnesses, adolescents work with their families and clinical professionals to help strengthen family relationships to better support the care of the child's illness. This involves group or individual meetings to discuss the best ways to support and talk about the child's health needs. This is something that may be tried in the care of adolescent HIV.
In what ways do you think this could be helpful?
Would you like for your family to take part in this kind of program?
If not, why not?

6.6 Is there anything else that you think would have helped your child to stay in care?
(Probe regarding possible solutions to the barriers mentioned in 6.1)

END INTERVIEW HERE

Section 7. Retained in care
7.1 Has your child ever been on medications to treat HIV?
(If No) What were the reasons your child was not started on treatment? (Skip to question 7.3)
(If Yes) When did your child first start treatment?
If there was a gap between linkage to care and initiation of treatment, why did that occur? / Why was there a delay in starting medications?
Did your child ever stop treatment for a period of time? If so, why?

7.2 What challenges has your child had with taking medications?
(Probes: Does your child ever skip doses? If so, why do you think that is?
What complaints has your child had about the medications?
Do you think your child feels like (he/she) can talk with you about reasons why (he/she) has trouble taking medicines?

Do you think your child feels like (he/she) can talk with people at the clinic about reasons why (he/she) has trouble taking medicines?

Tell me about the last time your child was supposed to take medicines, but didn't.)

***If the adolescent has had viral load testing, review these results together**. If the adolescent has had a detectable viral load recently, but the caregiver denies any problems with the medicines, discuss further with the caregiver. Ask if the child might have missed more doses than the caregiver had recalled or realized. And if so, what reasons might have caused the child to miss doses?*

7.3 For people on treatment, there are different things have been tried to help them take their medicines well and stay in care. I would like to tell you about these things and hear whether or not you think they would be helpful to other adolescents like yours in Botswana.

<u>Enhanced Personal Contacts</u>: In some places, there are people at the clinics who call patients between clinic visits to remind them about appointments and remind them to take their medicines if they are on medicines. The patients meet these people when they come to their appointments. When patients miss appointments, they call them to help them figure out how they can get back to the clinic.
Do you think this type of program would have been good for you and your family?
Why or why not?

7.4 <u>Clinical Decision Support</u>: In some places, clinic staff don't call patients with reminders, but do call if there is a problem such as a missed appointment or lab results that show that the person isn't taking the medicines well.
Do you think this type of program would have been good for you and your family?
Why or why not?

7.5 <u>Peer Support</u>: In some places, young people with HIV are linked to peers with HIV through support groups or other activities.
Do you think this type of program would have been good for your child?
Why or why not?

7.6 <u>Cell Phone Reminders</u>: Do you think it would help your child to get cell phone reminders telling them to take medicines or come to appointments?
Why or why not?
(*If reminders are seen as helpful*): Who do you think should get cell phone reminders? (The child / the parent / both)

7.7 <u>Family Therapy</u>: For other illnesses, adolescents work with their families and clinical professionals to help strengthen family relationships to better support the care of the child's illness. This involves group or individual meetings to discuss the best ways to support and talk about the child's health needs. This is something that may be tried in the care of adolescent HIV.
In what ways do you think this could be helpful?
Would you like for your family to take part in this kind of program?

If not, why not?

7.8 Is there anything else that you think would help your child to get the HIV care that they need?

Patient Interview Guide – DISCLOSED

Section 1. Demographics

1.1 Orphan status: (maternal, paternal, both, neither)

1.2 Current occupation (month prior to hospitalization): (full-time student, part-time student, part-time work, full-time work, too ill to go to school or work, other)

1.3 Last completed school grade:

1.4 Did you ever have to either repeat a year of school or to stop attending school because you were frequently ill?
(If yes, provide details.
When did these illnesses occur? What were your symptoms? Were you hospitalized? What were the reasons you needed to postpone or stop your education?)

1.5 HIV status of biological mother (positive, negative, unknown)

1.6 Anyone in the home with HIV? (If yes, ask relation to patient)

Section 2. History of Present Illness / Past Medical History

2.1 Why are you in the hospital?
(Probe for details of what the adolescent understands about their current illness or hospitalization.
If patient's answer differs from registry and chart information, probe for clarification.)

2.2 How long have you been sick, or unwell?
(Probe for specifics about disease course:
Ask about symptoms, progression of illness since they first became sick.
Where did they go for care? (clinic / hospital / traditional healer)
What was done at these medical visits?)

2.3 Have you ever been admitted to the hospital in the past?
(If so, when did the admission(s) occur?
What was/were the reason(s) for the admission(s)?
How long were you in the hospital?
Did you fully recover, or were you persistently ill?
Were you admitted frequently to the hospital?)

2.4 In the last year, did you ever go to clinic because you were sick?
(If so, please tell me about that.
When did you go to clinic?
What symptoms were you having?

Specifically ask if there was any presentation with: flu-like symptoms, fever, decreased appetite, sore throat, malaise or feeling "unwell", rash, night sweats, sores, swollen or tender lymph nodes, muscle aches, and/or headache.
What care did you receive?
Did you recover? Or were you persistently ill?)

Section 3. HIV testing and follow-up history – All patients.

3.1 Were you ever tested for HIV prior to this hospitalization?
3.1b **How many times did you present to care (to a clinic or hospital) before you were tested for HIV**?
What were the reasons for presenting to care? (What symptoms or illnesses did you have?)
Where did you go for care?
Did you think your illness might be related to HIV?
Were you ever offered an HIV test?
Did you ever ask to be tested for HIV?

Review the adolescent's health card and clarify details of previous clinic visits or hospitalizations and illnesses. *Note if each episode was a clinic visit or hospitalization.*

Month/Year	***Hospital or Clinic***	***Reason for Visit or Admission***

(If NOT diagnosed until this admission, SKIP to section 4)

3.2 When were you diagnosed?
(What illnesses were you suffering from? What led to you being tested? Where were you tested?
Why do you think you weren't tested earlier?)

3.3 After you were diagnosed, did you ever go to a clinic for HIV care?
(*If NOT connected to a treatment center, SKIP to section 5*)
How soon after you were diagnosed did you go to HIV care?
If there was a significant gap between diagnosis and linkage to care, why did this occur?
Where do you go for care?
Have you ever gone anywhere else for your care?
If so, why did you change where you went for care?

3.4 When was the last time you went to a clinic for HIV care?

(If MORE than 6 months ago SKIP to section 6; if LESS than 6 months ago, SKIP to section 7)

In the past, have you ever stopped going to clinic for a period of time?

When was that? Why were you unable to go to clinic during that time?

What helped you get back to clinic?

Section 4. Not diagnosed.

4.1 Did you ever think about getting tested for HIV before this hospitalization?

(*Probes: Why, or why not?*
If you, your caregivers, or doctors might have thought about it, but did not test:
Why weren't you tested?
Do you know where you can go to get tested?
Would you feel comfortable going there to get tested?
Need for consent a barrier?
Cost a barrier?
Stigma/fear of knowing a barrier?
Any other reasons you did not get tested?)

4.2 Is there anything that would have made you get tested for HIV earlier?

4.3 Do you have suggestions for how healthcare workers can help other young people get tested before they get sick?

4.4 Where do you think tests should be offered?

(Probes:
What about in schools?
What about in people's homes through door to door testing?
What are some reasons this could be helpful?
What do you think might be some problems with this?)

END INTERVIEW HERE

Section 5. Diagnosed but not linked to care.

5.1 When you were diagnosed with HIV, what were you told about getting HIV care?

(Probes:
Was care offered at the same place where you were diagnosed?
Why didn't you or your family do what you were told to do to get HIV care?
Probes: transportation, convenience, expense, confidentiality, stigma, trust in medical system)

In some places, different things have been tried to help people with HIV get the care they need. I would like to tell you about these things and hear whether or not you think they would be helpful to other young people like you in Botswana?

5.2 Extended Counseling: In some places, there are long counseling sessions for people who just learned that they have HIV. The counseling sessions happen in the homes of people with HIV and happen every month for about 2 hours. They talk about lots of things related to HIV, including why medicines for HIV are important.
Do you think this type of counseling program would have been good for you and your family? Why or why not?

5.3 Case Management: In some places, people called "case managers" meet with HIV+ people in whatever place the HIV+ person and their family are most comfortable. This might be in their homes or the clinic or anywhere else. They help them to connect to helpful resources such as support groups and clinics. They also help them to think about other ways to reach goals that they have in life.
Do you think this type of program would have been good for you and your family?
Why or why not?

5.4 Is there anything else that you think would have helped you to link to care?
END INTERVIEW HERE

Section 6. Linked but not retained in care.
6.1 Why did you stop going back to the clinic for HIV care?
(Probes: cost, family beliefs, patient-provider relationship)

In some places, different things have been tried to help people with HIV stay in care. I would like to tell you about these things and hear whether or not you think they would be helpful to other young people like you in Botswana?

6.2 Enhanced Personal Contacts: In some places, there are people at the clinics who call patients between clinic visits to remind them about appointments. The patients meet these people when they come to their appointments. When patients miss appointments, they call them to help them figure out how they can get back to the clinic.
Do you think this type of program would have been good for you and your family?
Why or why not?

6.3 Clinical Decision Support: In some places, clinic staff don't call patients with reminders, but do call if there is a problem such as a missed appointment.
Do you think this type of program would have been good for you and your family?
Why or why not?

6.4 Peer Support: In some places, young people with HIV are linked to peers with HIV through support groups or other activities.
Do you think this type of program would have been good for you?
Why or why not?

6.5 Family Therapy: For other illnesses, adolescents work with their families and clinical professionals to help strengthen family relationships to better support the care of the child's illness. This involves group or individual meetings to discuss the best ways to support and talk

about the child's health needs. This is something that may be tried in the care of adolescent HIV.
In what ways do you think this could be helpful?
Would you like for your family to take part in this kind of program?
If not, why not?

6.6 Is there anything else that you think would have helped you to stay in care?
(Probe regarding possible solutions to the barriers mentioned in 6.1)

END INTERVIEW HERE

<u>Section 7. Retained in care</u>
7.1 Have you ever been on medications to treat HIV?
(If No) What were the reasons you were not started on treatment? (Skip to question 7.3)
(If Yes) When did you first start treatment?
If there was a gap between linkage to care and initiation of treatment, why did that occur? / Why was there a delay in starting medications?
Did you ever stop treatment for a period of time? If so, why?

7.2 What challenges have you had with taking medications?
(Probes: Do you ever skip doses? If so, why?
How do the medicines make you feel?
Do you feel like you can talk with people at the clinic about reasons why you have trouble taking medicines?
Tell me about the last time you were supposed to take medicines, but didn't.)

If the adolescent has had viral load testing, review these results together*. If the adolescent has had a detectable viral load recently, but denies any problems with the medicines, discuss further with the patient. Ask if there might be more missed doses than they had recalled or realized. And if so, what reasons might have caused them to miss doses?*

7.3 For people on treatment, there are different things have been tried to help them take their medicines well and stay in care. I would like to tell you about these things and hear whether or not you think they would be helpful to other young people like you in Botswana.

<u>Enhanced Personal Contacts</u>: In some places, there are people at the clinics who call patients between clinic visits to remind them about appointments and remind them to take their medicines if they are on medicines. The patients meet these people when they come to their appointments. When patients miss appointments, they call them to help them figure out how they can get back to the clinic.
Do you think this type of program would have been good for you and your family?
Why or why not?

7.4 <u>Clinical Decision Support</u>: In some places, clinic staff don't call patients with reminders, but do call if there is a problem such as a missed appointment or lab results that show that the person isn't taking the medicines well.

Do you think this type of program would have been good for you and your family?
Why or why not?

7.5 Peer Support: In some places, young people with HIV are linked to peers with HIV through support groups or other activities.
Do you think this type of program would have been good for you?
Why or why not?

7.6 Cell Phone Reminders: Do you think it would help you to get cell phone reminders telling you to take medicines or come to appointments?
Why or why not?

7.7 Family Therapy: For other illnesses, adolescents work with their families and clinical professionals to help strengthen family relationships to better support the care of the child's illness. This involves group or individual meetings to discuss the best ways to support and talk about the child's health needs. This is something that may be tried in the care of adolescent HIV.
In what ways do you think this could be helpful?
Would you like for your family to take part in this kind of program?
If not, why not?

7.8 Is there anything else that you think would help you to get the HIV care that you need?

Patient Interview Guide – NOT DISCLOSED

Section 1. Demographics

1.1 Orphan status: (maternal, paternal, both, neither)

1.2 Current occupation (month prior to hospitalization): (full-time student, part-time student, part-time work, full-time work, too ill to go to school or work, other)

1.3 Last completed school grade:

1.4 Did you ever have to either repeat a year of school or to stop attending school because you were frequently ill?
(If yes, provide details.
When did these illnesses occur? What were your symptoms? Were you hospitalized? What were the reasons you needed to postpone or stop your education?)

Section 2. HPI/PMHx

2.1 Why are you in the hospital?
(Probe for details of what the adolescent understands about their current illness or hospitalization.
If patient's answer differs from registry and chart information, probe for clarification.)

2.2 How long have you been sick, or unwell?
(Probe for specifics about disease course:
Ask about symptoms, progression of illness since they first became sick.
Where did they go for care? (clinic / hospital / traditional healer)
What was done at these medical visits?)

2.3 Have you ever been admitted to the hospital in the past?
(If so, when did the admission(s) occur?
What was/were the reason(s) for the admission(s)?
How long were you in the hospital?
Did you fully recover, or were you persistently ill?
Were you admitted frequently to the hospital?)

2.4 In the last year, did you ever go to clinic because you were sick?
(If so, please tell me about that.
When did you go to clinic?
What symptoms were you having?
Specifically ask if there was any presentation with: flu-like symptoms, fever, decreased appetite, sore throat, malaise or feeling "unwell", rash, night sweats, sores, swollen or tender lymph nodes, muscle aches, and/or headache.
What care did you receive?
Did you recover? Or were you persistently ill?)

WITHOUT DISCLOSING STATUS, Review the adolescent's health card and clarify details of previous clinic visits or hospitalizations and illnesses. *Note if each episode was a clinic visit or hospitalization.*

Month/Year	***Hospital or Clinic***	***Reason for Visit or Admission***

2.5 Why do you think you are sick?

(If patient reveals that they know their status: How do you feel about it? What conversations have you had with your parent(s) about it? If patient is comfortable and agreeable to talking about their status, SKIP to ***DISCLOSED patient interview guide****, question 1.5)*

2.6 Do you take medications every day?

(If yes, What do the medicines do?
Probe for medicine names.)

2.7 What challenges have you had with taking medications?

(*Probes: Do you ever skip doses? If so, why?*
How do the medicines make you feel?
Do you feel like you can talk with people at the clinic about reasons why you have trouble taking medicines?
Tell me about the last time you were supposed to take medicines, but didn't.)
